# The complete chloroplast and mitochondrial genomes of Hyunsasi tree, *Populus alba* x *Populus glandulosa* (Salicaceae)

**DOI:** 10.1080/23802359.2019.1598788

**Published:** 2019-07-12

**Authors:** Jongsun Park, Yongsung Kim, Hong Xi, Woochan Kwon, Mi Kwon

**Affiliations:** a InfoBoss Co., Ltd., Seoul, Republic of Korea;; b InfoBoss Research Center, Seoul, Republic of Korea

**Keywords:** *Populus alba* x *Populus glandulosa*, chloroplast genome, mitochondrial genome, Salicaceae, Hyunsasi

## Abstract

*Populus alba* x *Populus glandulosa* is a hybrid species made by Dr. Hyun. It is called as Hyunsasi tree in Korea. It has been used as a good resource for genetic engineering because it is sterile. Here, we presented its complete chloroplast and mitochondrial genomes: chloroplast genome is identical to that of *P. alba* (156,505 bp and 129 genes covering 84 protein-coding genes, 37 tRNAs, and eight rRNAs) and mitochondrial genome is 813,261 bp long and GC ratio is 44.9%. It contains 59 genes containing 32 protein-coding genes, 22 tRNAs, and three rRNAs. Based on alignment with *P. tremula* x *P. alba* mitochondrial genome, 1,752 single nucleotide polymorphisms (SNPs; 0.22%) and 41,506 insertion and deletions (INDELs; 5.10%) are found. Phylogenetic trees based on chloroplast and mitochondrial genomes show different topology in four *Populus* species, indicating that both genomes may be evolved in different ways after common ancestors of *Populus* genus.

*Populus alba* x *Populus glandulosa*, called as En-su-won-sa-si tree in Korean, is hybrid species between *P. alba* and *P. glandulosa* made by Dr. Hyun in Korea (Son 2009). Because it contributed to recover bare mountains in Korea due to low level of economy more than fifty years ago, name was changed to Hyun-sa-si following his last name (Son 2009). This tree has been used for molecular biology because *Populus* is a model tree and it is sterile naturally blocking spreading inserted genes, so that it been studied intensively (Lee et al. 2005, 2007; Kim et al. 2012, 2013). To understand genetic background of Hyunsasi, we completed its chloroplast and mitochondrial genomes derived from its whole genome project (InfoBoss Co., Ltd.).

Total DNA of *P. alba x P. glandulosa* isolated from forest of National Institute of Forest Science, Seoul, Republic of Korea (Voucher in InfoBoss Cyber Herbarium (IN); IB-00597) was extracted from fresh leaves using a DNeasy Plant Mini Kit (QIAGEN, Hilden, Germany). Genome sequencing was performed using HiSeq2000 at Macrogen Inc., Korea, and *de novo* assembly and confirmation were completed using Velvet 1.2.10 (Zerbino and Birney 2008), SOAPGapCloser 1.12 (Zhao et al. 2011), BWA 0.7.17 (Li 2013), and SAMtools 1.9 (Li et al. 2009). Geneious R11 11.0.5 (Biomatters Ltd, Auckland, New Zealand) was also used for annotation of mitochondrial and chloroplast genomes based on *Populus tremula* x *Populus alba* mitochondrial genome (NC_028329; Mader et al. 2016) and *P. alba* chloroplast genome (NC_008235; Okumura et al. 2006), respectively.

Chloroplast genome of Hyunsasi (Genbank accession is MK358444) is identical to that of *P. alba* (156,505 bp and 129 genes covering 84 protein-coding genes, 37 tRNAs, and eight rRNAs; Okumura et al. 2006). Its mitochondrial genome (Genbank accession is MK358443) is 813,261 bp and overall GC content is 44.9%, similar to those of *P. tremula* x *P. alba* (783,513bp and 44.7%; Mader et al. 2016). It contains 59 genes (32 protein-coding genes, 22 tRNAs, and three rRNAs), which is same to that of *P. tremula* x *P. alba* (Mader et al. 2016) and less than one protein-coding gene by *Arabidopsis thaliana* (Unseld et al. 1997).

Based on alignment with mitochondrial genome of *P. tremula* x *P. alba*, a large amount of sequence variations is identified: 1,752 single nucleotide polymorphisms (SNPs; 0.22%) and 41,506 insertion and deletions (INDELs; 5.10%); while 11 non-synonymous SNPs and one insertion are found in 10 genes and one synonymous SNP is identified in nad2 gene (0.03% sequence variations). Four big insertion areas (33,848 bp in total) on Hyunsasi mitochondrial genomes are found at 76,999 bp to 77,894 bp, 496,836 bp to 27,683 bp, 548,818 bp to 547,297 bp, and 566,825 bp to 567,406 bp.

Four *Populus* and two complete chloroplast and mitochondrial genomes were aligned using MAFFT 7.388 (Katoh and Standley 2013), separately for constricting neighbor joining (bootstrap repeat is 10,000) and maximum likelihood (bootstrap repeat is 1,000) trees using MEGA X (Kumar et al. 2018). Both phylogenetic trees show different topology in four *Populus* species (Figure 1), reflecting that both genomes may be evolved in different ways after common ancestors of *Populus* genus.

**Figure 1.**
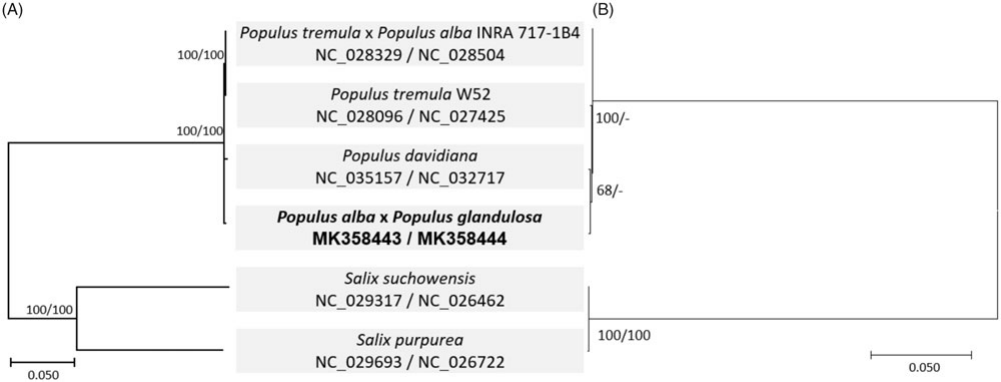
Two phylogenetic trees (A is phylogenetic tree based on mitochondrial genomes and B is phylogenetic tree based on chloroplast genomes) of neighbor joining (bootstrap repeat is 10,000) and maximum likelihood (bootstrap repeat is 1,000) based on four *Populus* and two *Salix* complete chloroplast and mitochondrial genomes: *Populus alba* x *Populus glandulosa* (mitochondrial genome is MK358443 and chloroplast genome is MK358444, in this study), *P. tremula* x *P. alba* (mitochondrial genome is NC_028329 and chloroplast genome is NC_028504), *Populus davidiana* (mitochondrial genome is NC_035157 and chloroplast genome is NC_032717), *Populus tremula* W52 (mitochondrial genome is NC_028096 and chloroplast genome is NC027425), *Salix suchowensis* (mitochondrial genome is NC_029317 and chloroplast genome is NC_026462), and *Salix purpurea* (mitochondrial genome is NC_029693 and chloroplast genome is NC_026722). The numbers above branches indicate bootstrap support values of neighbor joining and maximum likelihood phylogenetic trees, respectively.
